# HOXB9 mediates resistance to chemotherapy and patient outcomes through the TGFβ pathway in pancreatic cancer

**DOI:** 10.18632/oncotarget.28235

**Published:** 2022-05-25

**Authors:** Naokazu Chiba, Shigeto Ochiai, Takahiro Gunji, Toshimichi Kobayashi, Toru Sano, Koichi Tomita, Shigeyuki Kawachi

**Affiliations:** ^1^Department of Digestive and Transplantation Surgery, Tokyo Medical University Hachioji Medical Center, Hachioji, Tokyo, Japan

**Keywords:** HOXB9, pancreatic cancer, epithelial mesenchymal transition, TGFβ

## Abstract

Background: Although HOXB9 induces tumor proliferation and chemoresistance in several cancer cells, little is known in pancreatic ductal adenocarcinoma (PDAC). In the present study, increased expression of HOXB9 in PDAC was associated with the induction of angiogenic factors and poor overall survival through the TGFβ pathway. Taken together, these results suggested that HOXB9 expression in PDAC could be a surrogate marker in clinical treatment.

Methods: *In vitro*, angiogenic factors, TGFβ signature, Epithelial Mesenchymal Transition (EMT) marker, and chemoresistance were examined in PDAC cell lines by HOXB9 knockdown system. And the reverse effect was confirmed by using TGFβ1 recombinant. Furthermore, in clinical specimens, the correlation between HOXB9 expression and TGFβ signature was analyzed, and the relationship with clinical outcomes were investigated.

Results: HOXB9 expression regulated the expression of TGFβ1 signature, angiogenic factors, and EMT markers *in vitro*, and TGFβ1 recombinant made the reverse effect of these results. And HOXB9 expression regulated the resistance to chemotherapy (Gemcitabine and nab-Paclitaxel) and stem cell population. Moreover, increased HOXB9 expression was significantly associated with poor disease-free survival the prognosis for overall survival. And, a significant positive correlation was observed between HOXB9 expression and several TGFβ signatures in clinical specimens.

Conclusions: In conclusion, HOXB9 expression could mediate angiogenesis, EMT, and cancer stemness through the TGFβ pathway, thereby resulting in chemoresistance and poor overall outcomes in patients with pancreatic cancer. Our results suggested that HOXB9 may clinically serve as a novel surrogate biomarker.

## INTRODUCTION

HOXB9, a member of the class I homeobox (HOX) genes, regulates various cellular signal transductions, including epithelial-mesenchymal transition (EMT), angiogenesis, and the maintenance of cell fate [1]. An increasing number of studies demonstrate that the HOXB9 gene is associated with cancer progression and resistance to chemoradiation [2, 3]. HOXB9 controls erythroblastic leukemia viral oncogene homolog (ErbB), TGFb pathway, and angiogenic factor, resulting in EMT, as well as tumor proliferation and chemoresistance; however, little is known about the expression of HOXB9 in pancreatic ductal adenocarcinoma (PDAC).

PDAC is the fourth-leading cause of cancer death and is known that five-year overall survival rate is about 5% [4]. Pancreatic cancer has invasion to surrounding organs at an early stage, causing distant metastasis [5]. Therefore, various pathological and molecular efforts have been made to improve postoperative progression and prognosis of PDAC.

In this study, it was clarified that the expression of HOXB9 causes enhancement of angiogenic factors and chemoresistance in patients with PDAC, and as a result, contributes to poor prognosis. HOXB9 could upregulate angiogenic factors and TGF signatures *in vitro*, and induce EMT and chemoresistance, upon reversal of effect by TGFβ recombinant. Taken together, these results suggested that HOXB9 expression in PDAC could be a surrogate marker in guiding clinical treatment.

## RESULTS

### HOXB9 expression in PDAC

**Figure 1:**
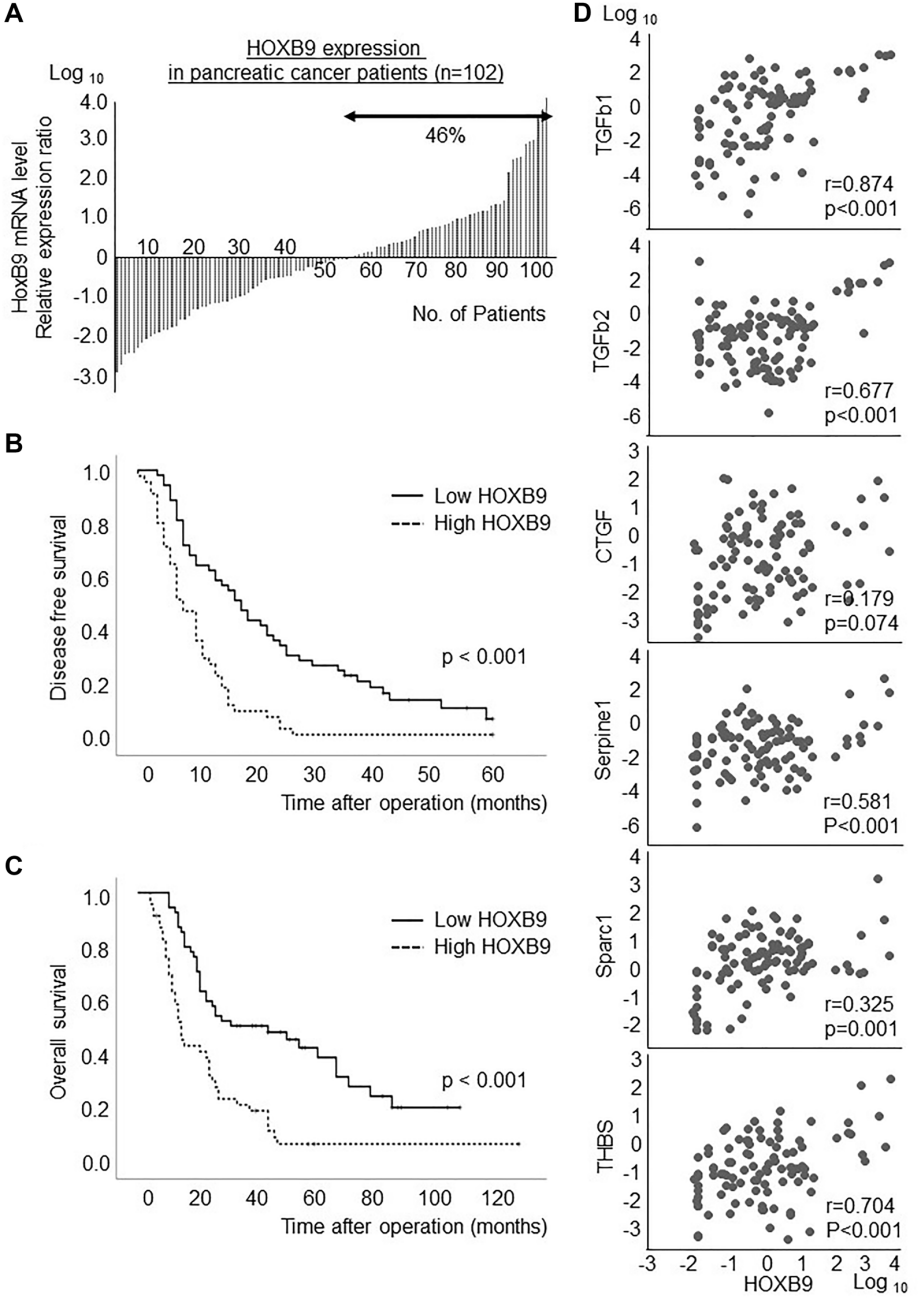
HOXB9 expression in PDAC patients. HOXB9 mRNA was highly expressed in approximately 46% of the patients (**A**). Increased HOXB9 expression was significantly associated with poor disease-free survival (*p* < 0.001) (**B**) and the prognosis for overall survival (*p* < 0.001) (**C**). Moreover, a significant positive correlation was observed between HOXB9 expression and several TGFβ signatures (**D**).

The HOXB9 expression was examined in the samples from 102 patients with PDAC who underwent curative resection at our institute. HOXB9 mRNA was highly expressed in approximately 46% of the patients (Figure 1A). Specific patients’ data including clinical and pathological factors was showed in the Supplementary Table 1. Increased HOXB9 expression was significantly associated with poor disease-free survival (*p* < 0.001) (Figure 1B) and the prognosis for overall survival (*p* < 0.001) (Figure 1C), as previously reported for hepatocellular carcinoma [6]. Moreover, a positive correlation was significantly observed between HOXB9 expression and several TGFβ signatures (Figure 1D).

### HOXB9 regulates the expression of TGFβ1 signature, angiogenic factors, and EMT markers

**Figure 2:**
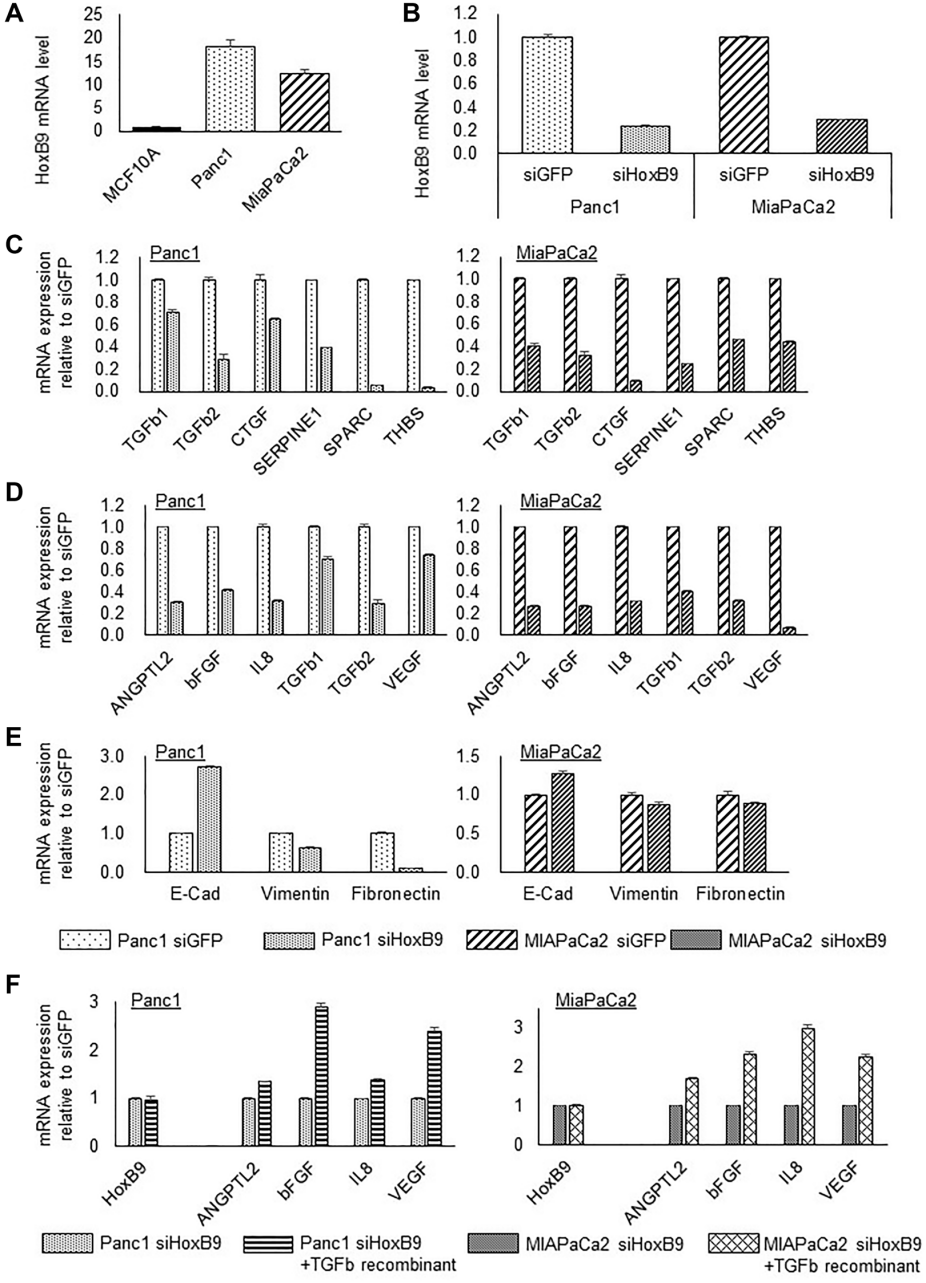
HOXB9 regulates the expression of TGFβ1 signature, angiogenic factors, and EMT markers. PDAC cell lines, Panc1 and MiaPaCa2, showed high HOXB9 mRNA expression relative to the human mammary epithelial cell line, MCF10A (**A**). siRNAs substantially reduced HOXB9 expression in PDAC cells by approximately 80% (**B**). HOXB9 expression by these siRNAs also correlated to the reduction in the expression of several TGFβ1 signatures (**C**) and angiogenic factors (**D**). In addition, reduction in HOXB9 expression in the two PDAC cell lines also mitigated EMT (**E**). Moreover, the angiogenic factors reduced upon HOXB9 depletion were amplified by incubation with the TGFβ1 recombinant construct (**F**).

PDAC cell lines, Panc1 and MiaPaCa2, showed high HOXB9 mRNA expression relative to the human mammary epithelial cell line, MCF10A, having low HOXB9 mRNA expression as reported previously (Figure 2A) [6]. In order to examine the effect of HOXB9 attenuation, the siHOXB9 knockdown system was performed *in vitro*. Both siRNAs substantially reduced HOXB9 expression in PDAC cells by approximately 80 % (Figure 2B). HOXB9 expression downregulated by these siRNAs also correlated to the reduction in the expression of several TGFβ1 signatures (Figure 2C) and angiogenic factors (Figure 2D). In addition, reduction in HOXB9 expression in the two PDAC cell lines also mitigated EMT, and several EMT markers showed a tendency for mesenchymal-epithelial transition (MET) (Figure 2E). Moreover, the angiogenic factors reduced upon HOXB9 depletion were amplified by incubation with the TGFβ1 recombinant construct (Figure 2F).

### HOXB9 regulates resistance to chemotherapy and stem cell population

**Figure 3:**
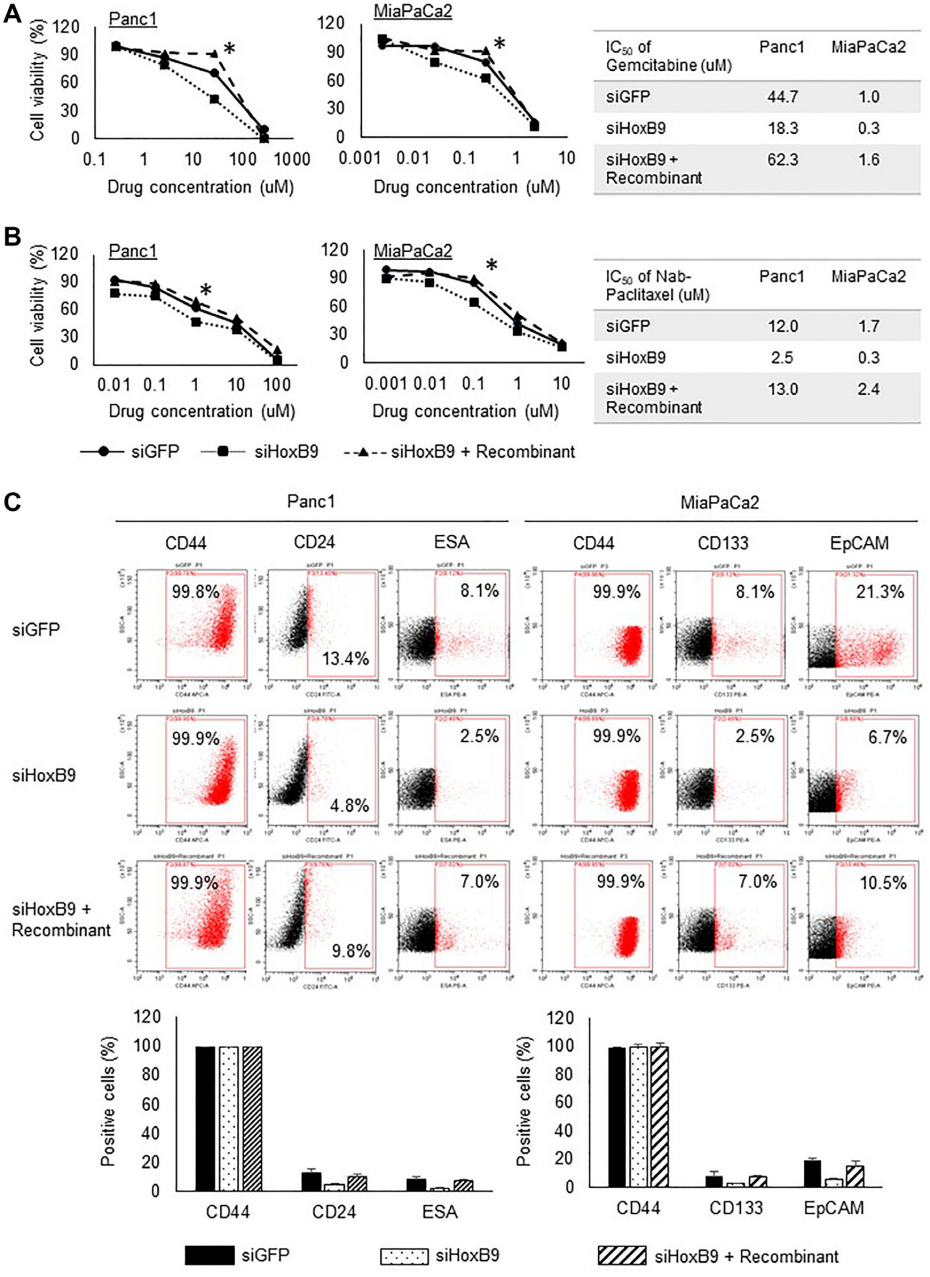
HOXB9 regulates resistance to chemotherapy and stem cell population. HOXB9-knockdown in Panc1 and MIApaCa2 cell lines elevated the sensitivity of these cells to gemcitabine and nab-paclitaxel treatment; the HOXB9 knockdown effect was rescued by TGFβ1 recombinant (**A**, **B**). An asterisk marking is added to the values that are statistically significantly different from siGFP. The IC50 values of gemcitabine and nab-paclitaxel were significantly reduced in HOXB9-knocked down Panc1 and MIAPaCa2 cells (right panel of A and B). As shown in (**C**, left panel), CD44 was highly expressed in all cells, however, the expressions of CD24 and ESA were reduced upon HOXB9 knockdown; these were restored to control levels upon TGFβ1 recombinant treatment. As shown in (C, right panel), CD44 was highly expressed in all cells, however, the expressions of CD133 and EpCAM were reduced upon HOXB9 knockdown; these returned to control levels upon TGFβ1 recombinant treatment.

To examine the association between HOXB9 expression and chemoresistance to gemcitabine and nab-paclitaxel in the PDAC cells, the toxicity of gemcitabine was measured using the MTT assay. HOXB9-knockdown in Panc1 and MIApaCa2 cell lines elevated the sensitivity of these cells to gemcitabine and nab-paclitaxel treatment; the HOXB9 knockdown effect was rescued to the sensitivity level in control cells upon treatment with the TGFβ1 recombinant (Figure 3A and 3B). The IC50 values of gemcitabine and nab-paclitaxel were significantly reduced in HOXB9-knocked down Panc1 and MIAPaCa2 cells (right panel of Figure 3A and 3B).

We characterized the Panc1 cancer stem cells through the expression of canonical surface stem cell markers, namely CD44, CD24, and ESA. The percentages of cells expressing CD44, CD24, and ESA were determined in siGFP, siHOXB9, and siHOXB9 with TGFβ1 recombinant conditions. As shown in Figure 3C (left panel), CD44 was highly expressed in all cells, however, the expressions of CD24 and ESA were reduced upon HOXB9 knockdown; these were restored to control levels upon TGFβ1 recombinant treatment. We also characterized the MIAPaCa2 cancer stem cells for the expression of the typical surface stem cell markers, namely CD44, CD133, and EpCAM. The percentages of cells expressing CD44, CD133, and EpCAM were determined in siGFP, siHOXB9, and siHOXB9 with TGFβ1 recombinant conditions. As shown in Figure 3C right panel, CD44 was highly expressed in all cells, however, the expressions of CD133 and EpCAM were reduced upon HOXB9 knockdown; these returned to control levels upon TGFβ1 recombinant treatment, similar to the findings in the Panc1 cells.

## DISCUSSION

HOX family of proteins function as monomers or homodimers and regulate the transcription of downstream genes directly, thereby controlling various cell functions, including differentiation, apoptosis, cell motility, and angiogenesis [7, 8]. In particular, as a member of the HOX family of transcription factors, HOXB9 is crucial for thoracic skeletal element specification and mammary gland development [9, 10]. In addition to regulating important developmental functions, HOXB9 has been reported to be elevated in various tumor cells, including colorectal cancer, breast cancer, and hepatocellular carcinoma, wherein increased HOXB9 expression is a predictor of poor outcomes [2, 3, 6, 11, 12]. From a biological perspective, HOX-Biding site is known to exist in the promoter region of angiopoietin-like 2, IL8, TGF-β2, VEGF, and bFGF. AREG, ERG, VEGF, and bFGF. In the present study, we demonstrated that HOXB9 could promote the expression of angiogenic factors and EMT in pancreatic cancer through the TGFβ pathway. Signaling through the TGFβ cascade plays a critical role in the development of pancreatic cancer and may lead to tumorigenesis, EMT, enhanced stemness in cancer cells, and poor overall survival in patients with pancreatic cancer.

In this study, the hypothesis is that TGFβ signaling is an important signaling component related to the HOXB9 gene functions; all experiments were performed accordingly. TGFβ triggered an increase in the expression of mesenchymal markers and a concomitant decrease in that of the epithelial markers. TGFβ also constructs a preferential environment for the phenotypic transition and induces invasiveness in cancer cells. TGFβ stimulation causes the phosphorylation of Smad2 and Smad3 on the SSXS motif in the C-terminal residues, which results in the formation of a complex with the Smad4 common mediator and subsequent nuclear translocation [13]. The results showed that HOXB9 could strongly up-regulate the TGFβ cascade in the human pancreatic cancer cell lines, and the TGFβ recombinant induction contributed significantly to the reversal of trends observed in the siHOXB9 experiments. Thus, the regulation of TGFβ signaling in HOXB9 may be an important pharmacological pathway underlying the invasion and metastasis of pancreatic cancer cells.

Various surface markers for pancreatic cancer stem cell-like populations have been reported, however, their utility in determining cancer stemness in pancreatic cancer remains unknown [14, 15]. In this study, surface marker expressions for the pancreatic cancer stem cell-like population, namely CD44, CD24, and ESA for Panc1 cells and CD44, CD133, and EpCAM in MiaPaCa2 cells were investigated. Induction of HOXB9 expression with or without recombinant TGFβ showed variations for these cell surface marker expressions in certain cell populations. HOBX9 expression may increase the cancer stem cell-like population.

Gemcitabine and nab-paclitaxel are widely used as first-line chemotherapeutic agents for the treatment of patients with pancreatic cancer. However, Various factors are intertwined in the mechanism of chemoresistance, and it is very difficult to understand clearly. Recent reports suggest that the tumor microenvironment plays a very important role in chemoresistance, wherein TGFβ signaling is important for the maintenance of healthy cells and it may regulate cancer invasion and metastasis, as well as chemoresistance through EMT [16, 17]. Our data suggested that TGFβ signaling induced by HOXB9 expression resulted in chemoresistance in pancreatic cancer cells through EMT.

We did not perform *in vivo* experiments but instead used the data obtained from resected specimens. The TGFβ signature and surgical outcomes were examined in clinical specimens. *In vivo* verification is also required in the future, however, we believe that the results from the clinical specimens substantially support the *in vitro* findings.

In conclusion, HOXB9 expression could mediate angiogenesis, EMT, and cancer stemness through the TGFβ pathway, thereby resulting in chemoresistance and poor overall outcomes in patients with pancreatic cancer. Our results suggested that HOXB9 may serve as a novel biomarker for selecting patients with pancreatic cancer who are more likely to benefit from chemotherapy and surgery.

## MATERIALS AND METHODS

### Cell lines and cell culture

The human PDAC cell lines, Panc1 and MiaPaCa2, were purchased from the American Type Culture Collection (ATCC, Manassas, VA, USA). The identity of all cells was independently confirmed by short tandem repeat genotyping in Sep 2021. Cell lines were cultured in RPMI 1640 medium (Sigma Chemical, St Louis, MO, USA) supplemented with 10% fetal bovine serum (FBS) at 37°C in a normoxic humidified atmosphere with 5% CO_2_. The cell lines were confirmed to be pathogen-free. The BIONIX-3 hypoxic culture kit (Sugiyama-Gen, Tokyo, Japan) was used to be cultured with hypoxic conditions.

### siRNA-mediated gene knock-down

Stealth™ RNAi siRNAs purchased from Invitrogen were used to knockdown the endogenous gene expression. Two siRNAs with different sequences of HOXB9 were independently transfected into cells at a final concentration of 100 nM using Lipofectamine RNAiMAX (Invitrogen). In the subsequent experiments, the siRNA sequence that could effectively suppress the target gene expression was used. siGFP was used as a negative control. These knockdown systems were verified by qRT-PCR. The sequences of all the primers used have been described previously [6, 18].

### Tissue samples

Frozen tissue specimens containing cancerous and correspondingly matched normal pancreas specimen were obtained from 102 patients who underwent surgical resection for pancreatic cancer between September 2008 and December 2013 at the Tokyo Medical University Hachioji Medical Center. All protocols were performed following the guidelines of the ethics committee of Tokyo Medical University.

The expression ratio (cancer to normal pancreatic cells surrounded by cancer cells) was examined using qRT-PCR. RNA was extracted from cells using the RNeasy kit (Qiagen, Valencia, CA, USA). Conditions for semi-quantitative amplification of cDNA were as follows: 95°C for 2 min, followed by 25 cycles of 95°C for 30 s, 56°C for 30 s, and 72°C for 60 s, with a final extension cycle of 72°C for 10 min. RT-PCR analysis was performed in triplicate for each sample on the Light Cycler 480 Real-Time PCR System using SYBR Green 1 Master Mix (Roche). The following program was run: pre-incubation for 5 min at 95°C, amplification for 45 cycles (10 s of denaturation at 95°C, 10 s of annealing at 57°C, and a 10-s extension at 72°C), for melt-curve analysis. These procedure of qRT-PCR is similar to that previously described articles [6, 18]. The mRNA levels of the target genes were normalized against the mRNA levels of GAPDH, which was used as an internal control. The sequences of all the primers used have been described previously [6, 18].

### TGFβ1 recombinant treatment

Cells were seeded on a 12- or 24-well culture plate or 10 cm^2^ dish and cultured for one day. TGFβ1 recombinant (H8541, Sigma-Aldrich, Germany) was transfected into the cells 48 h before extraction of mRNA and protein. The concentration of DMSO was finally adjusted to be 0.05% or less.

### MTT assay

Cells in the 96-well plates were treated with gemcitabine or nab-paclitaxel at final concentrations of 0.001–1000 μM for 72 h. Next, the MTT reagent (Sigma, St. Louis, MO, USA) was added, and the cells were cultured for 4 h before the absorbance was measured at 450 nm. Cell viability was calculated as the amount of MTT converted relative to the control cells without chemo reagent treatment.

### Fluorescence-activated single-cell sorting (FACS)

PANC-1 and MIA-PaCa-2 transfected cells were cultured in RPMI supplemented with 10% FBS before dissociation with trypsin solution. After dissociation into single cells, PANC-1 and MIA-PaCa-2 cells were washed with PBS and counted. The cells were then resuspended in incubation buffer (PBS supplemented with 3% FBS) at a final concentration of 1 × 10^7^ cells/mL. APC-conjugated anti-human CD44 (sc-7297 APC, Santa Cruz Biotechnology, Dallas, TX, USA), FITC-conjugated anti-human CD24 (sc-19585 FITC, Santa Cruz Biotechnology, Dallas, TX, USA), PE-conjugated anti-human ESA (sc-28320 PE, Santa Cruz Biotechnology, Dallas, TX, USA), PE-conjugated anti-human CD133 (sc-365537 PE, Santa Cruz Biotechnology, Dallas, TX, USA), and PE-conjugated anti-human EpCAM (sc-25308 PE; Santa Cruz Biotechnology, Dallas, TX, USA) were added to the samples according to the manufacturer’s instructions and incubated at 4°C in the dark. After 30 min of incubation, the cells were washed twice and analyzed on a flow cytometer (CytoFLEX, Beckman Coulter, Inc., Brea, CA, USA).

### Statistical analyses

Bars represent the mean ± standard error of the mean. Comparisons between groups were made using a two-tailed *t*- or *U*-test for continuous variables and the Fisher’s exact test for comparison of proportions. Correlations were calculated using the nonparametric Spearman coefficient. All calculations were performed using the SPSS software (SPSS 24.0). Overall survival (OS) curves were plotted according to the Kaplan–Meier estimates and compared for significance using log-rank tests. Statistical significance was set at *P* < 0.05. These statistical processing is the same as in the previous articles [6, 18].

## SUPPLEMENTARY MATERIALS



## Abbreviations

HOXB9: Homeobox B9
HOX: Homeobox
VEGF: Vascular endothelial growth factor
OS: Overall survival
siRNA: short interference RNA
qRT-PCR: quantitative reverse transcription- polymerase chain reaction

## References

[1] Cantile M , Schiavo G , Terracciano L , Cillo C . Homeobox genes in normal and abnormal vasculogenesis. Nutr Metab Cardiovasc Dis. 2008; 18:651–58. 10.1016/j.numecd.2008.08.001. 19013779

[2] Hayashida T , Takahashi F , Chiba N , Brachtel E , Takahashi M , Godin-Heymann N , Gross KW , Vivanco MD , Wijendran V , Shioda T , Sgroi D , Donahoe PK , Maheswaran S . HOXB9, a gene overexpressed in breast cancer, promotes tumorigenicity and lung metastasis. Proc Natl Acad Sci U S A. 2010; 107:1100–5. 10.1073/pnas.0912710107. 20080567 PMC2824265

[3] Chiba N , Comaills V , Shiotani B , Takahashi F , Shimada T , Tajima K , Winokur D , Hayashida T , Willers H , Brachtel E , Vivanco MD , Haber DA , Zou L , Maheswaran S . Homeobox B9 induces epithelial-to-mesenchymal transition-associated radioresistance by accelerating DNA damage responses. Proc Natl Acad Sci U S A. 2012; 109:2760–65. 10.1073/pnas.1018867108. 21930940 PMC3286905

[4] Siegel R , DeSantis C , Virgo K , Stein K , Mariotto A , Smith T , Cooper D , Gansler T , Lerro C , Fedewa S , Lin C , Leach C , Cannady RS , et al. Cancer treatment and survivorship statistics, 2012. CA Cancer J Clin. 2012; 62:220–41. 10.3322/caac.21149. 22700443

[5] Li D , Xie K , Wolff R , Abbruzzese JL . Pancreatic cancer. Lancet. 2004; 363:1049–57. 10.1016/S0140-6736(04)15841-8. 15051286

[6] Chiba N , Ozawa Y , Hikita K , Okihara M , Sano T , Tomita K , Takano K , Kawachi S . Increased expression of HOXB9 in hepatocellular carcinoma predicts poor overall survival but a beneficial response to sorafenib. Oncol Rep. 2017; 37:2270–76. 10.3892/or.2017.5474. 28260092

[7] Grier DG , Thompson A , Kwasniewska A , McGonigle GJ , Halliday HL , Lappin TR . The pathophysiology of HOX genes and their role in cancer. J Pathol. 2005; 205:154–71. 10.1002/path.1710. 15643670

[8] Chen H , Sukumar S . HOX genes: emerging stars in cancer. Cancer Biol Ther. 2003; 2:524–25. 10.4161/cbt.2.5.525. 14614319

[9] Fromental-Ramain C , Warot X , Lakkaraju S , Favier B , Haack H , Birling C , Dierich A , Doll EP , Chambon P . Specific and redundant functions of the paralogous Hoxa-9 and Hoxd-9 genes in forelimb and axial skeleton patterning. Development. 1996; 122:461–72. 10.1242/dev.122.2.461. 8625797

[10] Chen F , Capecchi MR . Paralogous mouse Hox genes, Hoxa9, Hoxb9, and Hoxd9, function together to control development of the mammary gland in response to pregnancy. Proc Natl Acad Sci U S A. 1999; 96:541–46. 10.1073/pnas.96.2.541. 9892669 PMC15172

[11] Seki H , Hayashida T , Jinno H , Hirose S , Sakata M , Takahashi M , Maheswaran S , Mukai M , Kitagawa Y . HOXB9 expression promoting tumor cell proliferation and angiogenesis is associated with clinical outcomes in breast cancer patients. Ann Surg Oncol. 2012; 19:1831–40. 10.1245/s10434-012-2295-5. 22396001

[12] Hoshino Y , Hayashida T , Hirata A , Takahashi H , Chiba N , Ohmura M , Wakui M , Jinno H , Hasegawa H , Maheswaran S , Suematsu M , Kitagawa Y . Bevacizumab terminates homeobox B9-induced tumor proliferation by silencing microenvironmental communication. Mol Cancer. 2014; 13:102. 10.1186/1476-4598-13-102. 24885802 PMC4023179

[13] Katsuno Y , Lamouille S , Derynck R . TGF-β signaling and epithelial-mesenchymal transition in cancer progression. Curr Opin Oncol. 2013; 25:76–84. 10.1097/CCO.0b013e32835b6371. 23197193

[14] Wang L , Li P , Hu W , Xia Y , Hu C , Liu L , Jiang X . CD44^+^CD24^+^ subset of PANC-1 cells exhibits radiation resistance via decreased levels of reactive oxygen species. Oncol Lett. 2017; 14:1341–46. 10.3892/ol.2017.6301. 28789349 PMC5529798

[15] Wei HJ , Yin T , Zhu Z , Shi PF , Tian Y , Wang CY . Expression of CD44, CD24 and ESA in pancreatic adenocarcinoma cell lines varies with local microenvironment. Hepatobiliary Pancreat Dis Int. 2011; 10:428–34. 10.1016/s1499-3872(11)60073-8. 21813394

[16] Pickup M , Novitskiy S , Moses HL . The roles of TGFβ in the tumour microenvironment. Nat Rev Cancer. 2013; 13:788–99. 10.1038/nrc3603. 24132110 PMC4025940

[17] Ma S , Qu W , Mao L , Zhu Z , Jia L , Zhao L , Zheng X . Antitumor effects of oncolytic adenovirus armed with Drosophila melanogaster deoxyribonucleoside kinase in colorectal cancer. Oncol Rep. 2012; 27:1443–50. 10.3892/or.2012.1665. 22294034

[18] Chiba N , Sunamura M , Nakagawa M , Koganezawa I , Yokozuka K , Kobayashi T , Hikita K , Ozawa Y , Okihara M , Sano T , Tomita K , Tsutsui R , Sugimoto M , Kawachi S . Overexpression of hydroxyproline via EGLN/HIF1A is associated with distant metastasis in pancreatic cancer. Am J Cancer Res. 2020; 10:2570–81. 32905516 PMC7471362

