# Supplementary material for: HOXB9 mediates resistance to chemotherapy and patient outcomes through the TGFβ pathway in pancreatic cancer
Source: Oncotarget. 2022 May 25;13:747–54. doi: 10.18632/oncotarget.28235 (PMC9132260; doi:10.18632/oncotarget.28235)
Supplement: Supplementary file 1 [file oncotarget-13-28235-s001.pdf]

HOXB9 mediates resistance to chemotherapy and patient outcomes through the TGFβ pathway in pancreatic cancer

SUPPLEMENTARY MATERIALS

Supplementary Table 1: Clinicopathological characteristics of 102 patients between high HOXB9 group and low HOXB9 group

	High HOXB9 (*n* = 47)	Low HOXB9 (*n* = 55)	*p*-value
Age	71 (17–84)	70 (48–84)	0.429
Gender			0.351
Male	23 (49%)	32 (58%)	
Female	24 (51%)	23 (42%)	
Procedure			0.561
PD or PPPD	37 (79%)	34 (41%)	
DP	8 (17%)	18 (33%)	
TP	2 (4%)	3 (6%)	
Final UICC stage			0.305
1	12 (26%)	15 (27%)	
2	27 (57%)	32 (58%)	
3	8 (17%)	8 (15%)	

Values without age are numbers. Values of age are median (range). Abbreviations: PD: pancreatoduodenectomy; PPPD: pylorus-preserving pancreatoduodenectomy; DP: distal pancreatectomy; TP: total pancreatectomy.
